# Public Mobility and Social Media Attention in Response to COVID-19 in Sweden and Denmark

**DOI:** 10.1001/jamanetworkopen.2020.33478

**Published:** 2021-01-04

**Authors:** Le Zhang, Isabell Brikell, Søren Dalsgaard, Zheng Chang

**Affiliations:** 1 Department of Medical Epidemiology and Biostatistics, Karolinska Institutet, Stockholm, Sweden; 2 National Centre for Register-based Research, Department of Economics and Business Economics, Aarhus University School of Business and Social Sciences Aarhus, Denmark

## Abstract

This ecological study evaluates the public mobility and social media attention associated with COVID-19 spread and societal interventions from February 15 to June 14, 2020, in Denmark and Sweden.

## Introduction

To reduce the spread of coronavirus disease 2019 (COVID-19), countries have implemented different societal interventions, and evaluation of the effect on public response is needed.^1^ Sweden and Denmark are comparable countries in terms of health care and sociodemographic characteristics; however, Denmark was one of the first countries to enforce lockdown and subsequent gradual reopening, whereas Sweden has had few restrictions, largely limited to public recommendations. We assessed public mobility and social media attention associated with COVID-19 spread and societal interventions from February 15 to June 14, 2020, in Denmark and Sweden.

## Methods

For this ecological study, public mobility was measured by Google mobility reports, providing daily percentage change in the number of visitors to public spaces compared with baseline (January 3 to February 6, 2020).^2^ We focused on mobility across retail and recreational spaces based on previous findings.^3^ Daily volume of tweets including COVID-19–related hashtags and keywords (eTable in the Supplement) were collected via Sprout Social to study public attention to the disease, capturing 732 634 tweets in Sweden and 324 730 in Denmark. Because this study used summary statistics rather than individual-level data, an institutional ethical review permit was not required according to the rules at Karolinska Institutet, Sweden. This study followed the part of the Strengthening the Reporting of Observational Studies in Epidemiology (STROBE) reporting guideline that are relevant to ecological studies.

The exposures that we focused on were disease spread (ie, daily new COVID-19 cases)^4^ and key societal interventions and announcements (ie, addresses by the prime minister or head of state) targeting public mobility. Interrupted time series analyses were conducted to evaluate the association between societal interventions and mobility, including both a level (β_1_) and a slope change (β_2_) that can be interpreted as the modeled percentage change in mobility from before to after the intervention.^5^ Statistical analyses were performed using R, version 3.6.3 (R Project for Statistical Computing). Details on measures and interrupted time series analyses are provided in the eMethods in the Supplement.

## Results

The percentage of mobility change, the number of COVID-19–related tweets, and the number of confirmed cases of COVID-19 are plotted with key societal interventions in the Figure. Daily volumes of COVID-19–related tweets followed a similar pattern in Denmark and Sweden, with volumes increasing exponentially during the beginning of disease spread and peaking between March 13 and 17 (from 746 on February 14 to 16 851 on March 13 in Sweden and from 25 to 8398 in Denmark). During this period, the World Health Organization declared a COVID-19 pandemic, Denmark announced a lockdown, and Sweden banned public gatherings or more than 500 people and recommended remote higher education and work. Twitter volumes then decreased with time in both countries. Public mobility to retail and recreation decreased in both countries during the study period, with a greater reduction in Denmark during lockdown. The maximum mean (SD) mobility reduction (apart from public holidays) in Denmark was 38% (2.6%) (from March 23 to 29) and 24% (2.1%) in Sweden (from March 30 to April 5). By the last week of the study period (June 8 to 14), mobility had returned to baseline levels in both countries, with a mean (SD) change of 2.14% (4.4%) in Denmark and −0.14% (5.1%) in Sweden (Figure).

**Figure.**
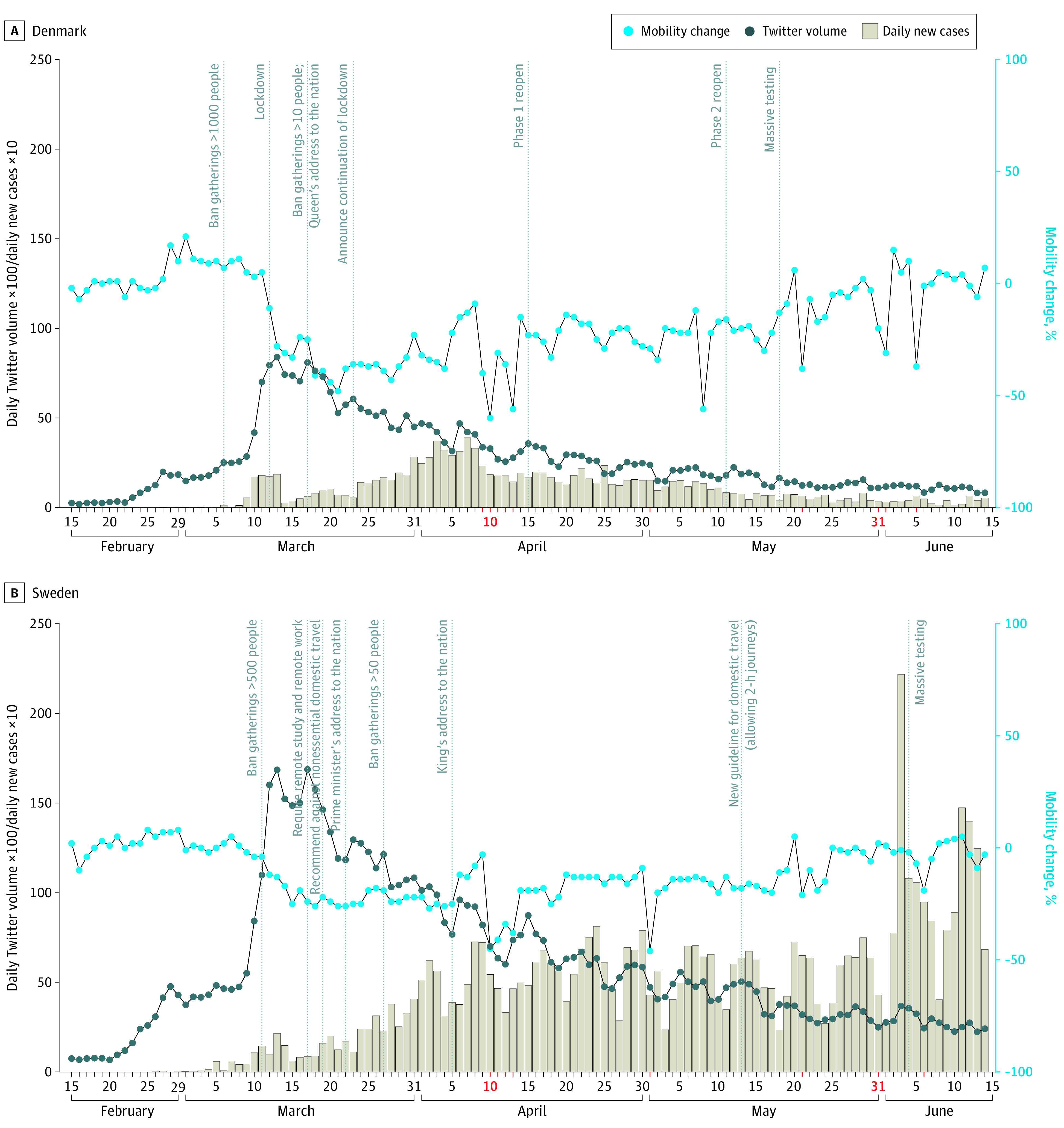
Mobility Change, Twitter Volume, and Spread of Coronavirus Disease 2019 (COVID-19) in Denmark and Sweden Public mobility was compared with the prepandemic baseline period (January 3 to February 6, 2020) across retail and recreational spaces (eg, restaurants, cafes, shopping centers, theme parks, museums, libraries, and movie theaters). Public holidays are marked in red.

The interrupted time series analyses showed that the Danish lockdown was associated with a 20% immediate decrease in mobility (β_1_, –20.19; 95% CI, –27.79 to –12.59) (Table). The ban of gatherings of more than 10 people coincided with the Queen’s address to the nation and was associated with a 12% decrease in mobility (β_1_, –12.20; 95% CI, –19.74 to –4.65). Phase 1 and 2 of the Danish reopening were associated with immediate reductions in mobility but increased mobility in the subsequent days. In Sweden, only the ban on public gatherings of more than 500 people was associated with a significant decrease in mobility (β_1_,–11.04; 95% CI, –14.66 to –7.42). Neither the prime minister’s nor the King’s address to the nation showed a statistically significant association with mobility. In both countries, higher numbers of daily confirmed COVID-19 cases were associated with reduced mobility (Denmark: β_1_,–2.66 [95% CI, –4.51 to –0.82]; Sweden: β_1_, –0.62 [95% CI, –0.84 to –0.40]).

**Table.** Associations of COVID-19 Interventions With Mobility Change in Denmark and Sweden

Intervention (date)	Level change β_1_ (95% CI)^a^	Slope change β_2_ (95% CI)^b^
**Denmark**		
Ban on gatherings of >1000 people (March 6)	6.98 (1.10 to 12.86)	–1.38 (–3.79 to 1.04)
Lockdown (March 12)	–20.19 (–27.79 to –12.59)	–2.05 (–3.87 to –0.24)
Ban on gatherings of >10 people and Queen’s address to the nation (March 17)	–12.20 (–19.74 to –4.65)	0.98 (–1.29 to 3.25)
Announcement of continuation of lockdown (March 23)	–0.05 (–6.46 to 6.36)	1.27 (0.99 to 1.55)
Phase 1 reopen (April 15)	–10.15 (–15.43 to –4.86)	0.06 (–0.13 to 0.25)
Phase 2 reopen (May 11)	–0.95 (–4.65 to 2.75)	0.70 (0.56 to 0.84)
Daily cases, per 100 persons	–2.66 (–4.51 to –0.82)	NA
**Sweden**		
Ban on gatherings of >500 people (March 11)	–11.04 (–14.66 to –7.42)	–1.99 (–3.14 to –0.83)
Requirement of remote study and remote work (March 17)	0 (–4.80 to 4.81)	NA
Recommendation against nonessential domestic travel (March 19)	0.42 (–3.96 to 4.80)	NA
Prime minister’s address to the nation (March 22)	–0.81 (–5.45 to 3.83)	1.79 (0.29 to 3.30)
Ban on gatherings of >50 people (March 27)	–3.99 (–8.69 to 0.71)	–0.06 (–0.68 to 0.56)
King’s address to the nation (April 5)	4.23 (0.60 to 7.85)	0.14 (0.05 to 0.24)
New guideline for domestic travel allowing 2-h journeys (May 13)	–0.53 (–2.89 to 1.83)	0.59 (0.48 to 0.69)
Daily cases, per 100 persons	–0.62 (–0.84 to –0.40)	NA

Abbreviations: COVID-19, coronavirus disease 2019; NA, not applicable.

^a^ Level change reflects the the gradual modeled percentage change in mobility change immediately after an intervention compared with preintervention.

^b^ Slope change reflects gradual modeled percentage change in mobility per day after an intervention until the next intervention, modeled only for interventions occurring at least 5 days apart. Negative estimates indicate reduced mobility; positive estimates, increased mobility.

## Discussion

In this cross-country comparison, stricter interventions were associated with larger reductions in mobility. Bans on public gatherings showed stronger associations with mobility in both countries compared with national announcements by country leaders and public recommendations, which were the primary intervention types in Sweden. We also found a delay in reaching maximum reduction in mobility after restrictions and for normalization of mobility after relaxation of restrictions, in line with prior research.^6^ Social media attention to COVID-19 on Twitter was greater for early interventions and disease spread than for later interventions and disease spread, with nearly identical patterns of decreasing COVID-19–related Twitter volumes across Denmark and Sweden despite marked difference in number of cases and the type of societal interventions. Better understanding of how interventions are associated with public mobility and attention may guide policy in relation to the resurgence of COVID-19 currently observed in many countries. Our findings are limited to short-term effects of specific interventions and do not capture interactions between these nor other important factors likely to be associated with public mobility and attention (eg, global media, guidelines from international health care organizations, and treatment and vaccine developments). Any conclusions are limited by the representativeness of Twitter and Goggle mobility data.

## Notes

Supplement. **eMethods.** **eTable.** English, Swedish and Danish COVID-19-related keywords and hashtags used for measuring twitter volume in Sweden and Denmark across the period February 15 to June 14, 2020
