# Supplementary material for: Public Mobility and Social Media Attention in Response to COVID-19 in Sweden and Denmark
Source: JAMA Netw Open. 2021 Jan 4;4(1):e2033478. doi: 10.1001/jamanetworkopen.2020.33478 (PMC7783540; doi:10.1001/jamanetworkopen.2020.33478)
Supplement: Supplement. — eMethods. eTable. English, Swedish and Danish COVID-19-related keywords and hashtags used for measuring twitter volume in Sweden and Denmark across the period February 15 to June 14, 2020 [file jamanetwopen-e2033478-s001.pdf]

Supplemental Online Content

Zhang L, Brikell I, Dalsgaard S, Chang Z. Public mobility and social media attention in response to COVID-19 in Sweden and Denmark. *JAMA Netw Open*. 2021;4(1):e2033478. doi:10.1001/jamanetworkopen.2020.33478

eMethods.

eTable. English, Swedish and Danish COVID-19-related keywords and hashtags used for measuring twitter volume in Sweden and Denmark across the period February 15 to June 14, 2020

This supplemental material has been provided by the authors to give readers additional information about their work.

© 2021 Zhang L et al. *JAMA Network Open.*

eMethods supplement

Measures

The outbreak of COVID-19 started in Denmark and Sweden around the 26-27 February 2020, and the two countries had similar initial trajectories of confirmed cases and mortality. Policy response however differed significantly across the two countries. Briefly, Denmark enforced a lockdown starting 11 March 2020, including closed educational facilities, restrictions on gatherings, various forms of business closure, limiting gatherings to > 10 persons. Restaurants were only allowed take-away, nightclubs, cinemas and concert venues were shut down and retail was restricted to high-street shops with malls being shut down entirely. This was followed by a gradual re-opening that in phase 1 largely affected educational facilities, and later the reopening of restaurants etc.[1] Sweden instead took a more lenient approach, with no national lockdown and interventions largely limited to recommendations (e.g. staying home at any sign of symptoms, limiting social interactions and use of public transport if possible). Physical distancing was strongly encouraged, but only mandatory in bars, restaurants, museums and events, which were allowed to remain open. Events >500 persons and then >50 were banned. Working from home was recommended, but only educational facilities for children aged 16 years or older closed for 3 months.[2] We utilize these divergent societal intervention strategies and differences in COVID-19 disease spread to study changes in public mobility and social media attention using below measures. We compiled societal interventions and news primarily from national public service media (Sveriges Television [SVT], Denmarks Radio [DR]) and official government websites (The Swedish Public Health Agency, the Danish Health Authority). We prioritized interventions and public announcements (i.e. address to the nation by the prime minister and head of state, a rare occurrence in Denmark and Sweden) aimed at reducing mobility.

Data on daily new confirmed COVID-19 cases were obtained from the Center for Systems Science and Engineering (CSSE) at Johns Hopkins University.[3] Data were accessed on July 27, 2020 and are thus subject to retrospective adjustments of reported number of cases.

Data on percent change in mobility across retail and recreational spaces were obtained from the "Google COVID-19 Community Mobility Reports", which are publically available and generated from aggregated, anonymized data from users who have turned on the 'Location History' setting on their mobile.[4,5] The data shows the percentage change of the number of visitors for a given weekday compared to a pre-COVID baseline, calculated as the median number of visitors for each weekday during January 3-February 6, 2020. Mobility is reported across six categories of places, including retail and recreation, groceries and

© 2021 Zhang L et al. *JAMA Network Open.*

pharmacies, parks, transit stations, workplaces, and residential. We focused on retail and recreational spaces (e.g. restaurants, cafes, shopping centers, theme parks, museums, libraries, and movie theaters) as it was found to display the greatest mobility change during the United Kingdom lockdown.[6] Further, it is more likely to reflect optional mobility. Finally, there was both cross-country and within-country variation in restrictions affecting spaces in this category (see above).

Twitter data was obtained through the official Twitter partner company Sprout Social. The daily volume of COVID-19 related tweets for Sweden and Denmark was collected by querying a list of COVID-19 related keywords and hashtags in both English, Swedish and Danish (eTable 1).

Statistical analysis

We used interrupted time-series (ITS) analysis[7] to evaluate how different societal interventions were associated with mobility change. We used a linear regression model to estimate the level change (i.e., mean mobility difference pre-intervention and post-intervention, β_1) and slope change (i.e. gradual change in mobility per day, β_2) associated with each intervention, separately for Sweden and Denmark. For Sweden, the interventions include ban gatherings >500 people, require remote study and remote work, recommend against non-essential domestic travel, prime minister's address to the nation, king's address to the nation, and new guideline for domestic travel (allowing 2 hours' journeys). For Denmark, the interventions include ban gatherings >1000 people, lockdown, ban gatherings >10 people, the queen's address to the nation, announced continuation of lockdown, phase one reopening and phase two reopening. As the slope change was intended to model the trend of mobility change over time, we excluded estimation of slope change for interventions occurring less than five days apart (this applies to "require remote study and remote work" and "recommend against non-essential domestic travel" in Sweden). In addition to the societal interventions, we included first-order autoregressive term of mobility change, daily confirmed cases (lag one day), national holidays and weekends as covariates.

© 2021 Zhang L et al. *JAMA Network Open.*

eReferences:

1. Andersen AL, Hansen ET, Johannesen N, Sheridan A. Pandemic, shutdown and consumer spending: Lessons from Scandinavian policy responses to COVID-19. *arXiv preprint arXiv:200504630.* 2020.
2. Ludvigsson JF. The first eight months of Sweden's COVID-19 strategy and the key actions and actors that were involved. *Acta Paediatrica.*n/a(n/a).
3. Dong E, Du H, Gardner L. An interactive web-based dashboard to track COVID-19 in real time. *The Lancet Infectious Diseases.* 2020;20(5):533-534.
4. Google LLC. "Google COVID-19 Community Mobility Reports". https://www.google.com/covid19/mobility/ Accessed November 14, 2020.
5. Aktay A, Bavadekar S, Cossoul G, et al. Google COVID-19 community mobility reports: Anonymization process description (version 1.1). *arXiv preprint arXiv:200404145.* 2020.
6. Drake TM, Docherty AB, Weiser TG, Yule S, Sheikh A, Harrison EM. The effects of physical distancing on population mobility during the COVID-19 pandemic in the UK. *The Lancet Digital Health.* 2020.
7. Bernal JL, Cummins S, Gasparrini A. Interrupted time series regression for the evaluation of public health interventions: a tutorial. *International journal of epidemiology.* 2017;46(1):348-355.

© 2021 Zhang L et al. *JAMA Network Open.*

eTable 1. English, Swedish and Danish COVID-19-related hashtags and keywords used for measuring twitter volume in Sweden and Denmark across the period February 15 to June 14, 2020

Keywords	Hashtags
Corona virus	#coronavirus
Coronavirus	#covid19
Covid-19	#COVID19dk
Covid19	#COVID19se
pandemic	#Coronakrise
epidemic	#Coronakrisen
coronasverige	#covid19danmark
Covid19sverige	#coronadanmark
coronadanmark	#covid19sverige
covid19Danmark	#coronasverige
COVID19dk	#Stannahemma
Covid19se	#Tvättahänderna
Coronakrise	#hållavstånd
Coronakrisen	#blivhjemme
Stanna hemma	#holdafstand
Tvätta händerna	#vaskhænder
Håll avstånd	#socialdistansering
Bliv hjemme	#socialafstand
Hold afstand	#isolering
Vask hænder	
social distansering	
social afstand	
Isolering	
pandemi	
utbrott	
smittan	
smitta	
sprids	
Immunitet	
Anders Tegnell	
udbrud	
smitte	
infektion	
spredning	
immun	
Søren Brostrøm	

© 2021 Zhang L et al. *JAMA Network Open.*
